# Pilot mental health during the COVID-19 pandemic: prevalence rates from semi-structured interviews, and associated vulnerability and protective factors

**DOI:** 10.3389/fpsyg.2023.1073857

**Published:** 2023-05-04

**Authors:** Corrie A. Ackland, Brett R. C. Molesworth, Jessica R. Grisham

**Affiliations:** ^1^School of Aviation, University of New South Wales, Sydney, NSW, Australia; ^2^School of Psychology, University of New South Wales, Sydney, NSW, Australia

**Keywords:** aviation, pilot, mental health, COVID-19, diagnoses

## Abstract

**Introduction:**

Pilots are a unique occupational group who perform a specialised job and face significant stressors. Pilot mental health has received increased attention since Germanwings Flight 9525; however, this research has largely focused on general anxiety, depression, and suicide and relied on a questionnaire-based methodology. This approach is likely to miss various mental health issues that may affect pilot wellbeing, leaving the prevalence of mental health issues in aviation unclear. In addition, the COVID-19 pandemic is likely to have a particular impact on the mental health and wellbeing of pilots, who experienced the devastating effect of COVID-19 on the industry.

**Method:**

In the present study, we conducted a comprehensive assessment of 73 commercial pilots during the COVID-19 pandemic, using the DIAMOND semi-structured diagnostic interview and explored possible associated vulnerability and protective factors, including life event stressors, personality, passion, lifestyle factors, and coping strategies.

**Results:**

The COVID-19 pandemic had a significant impact on aviation during the time of this study, affecting 95% of participants. The diagnostic results revealed over one third of pilots had symptoms of a diagnoseable mental health disorder. Anxiety disorders were the most commonly found disorders, followed by Attention Deficit Hyperactivity Disorder (ADHD), Adjustment Disorder, and Depressive Disorders. Pilots’ high life event scores placed them at an increased risk for the development of stress-related illness, though did not explain which pilots had mental health difficulties in this study. Regression analysis supported a diathesis-stress model for pilot mental health, with disagreeableness and obsessive passion contributing to pilots’ development of mental health issues, and nutrition as the most important protective factor.

**Discussion:**

This study, though limited to the COVID-19 pandemic, provides a valuable precedent for a more thorough assessment of pilot mental health, and contributes to the broader understanding of pilot mental health, including suggestions to target factors associated with the development of mental health issues.

## Introduction

This study aims to assess pilot mental health during the COVID-19 pandemic related industry disruption. It will achieve this aim through the use of a semi structured diagnostic interview, coupled with a battery of questionnaires. This study is important as previous studies have largely relied on questionnaire or database methodologies, which may not fully capture the entire state of pilot mental health (i.e., focus on depression and/or suicide; Ackland et al., 2022). Guided by the diathesis-stress model, this study will also investigate associated stressors, vulnerability, and protective factors.

Pilots perform a specialised job with unique stressors. As shift-workers and remote-workers, they suffer fatigue and circadian disturbance, and tolerate physiological stress incurred by noise, and cramped, sedentary working conditions (Cahill et al., 2019). In commercial roles, pilots are also responsible for their passengers’ safety and undergo frequent checking, training, and medical evaluations (Bor et al., 2017). Such factors are sources of pilot work-related stress and have the potential to adversely affect pilot mental health (Cahill et al., 2019).

Previous studies of pilot mental health have found that pilots suffer from rates of depression and suicidal thoughts comparable to, or exceeding that of the general population (Wu et al., 2016; Cahill et al., 2019). Few studies have assessed broader mental health issues in the pilot population, possibly due to, and also perpetuating the assumption that depression and suicidality is the most severe and concerning mental health issue for pilots.

However, although events such as the Germanwings Flight 9525, in which the copilot deliberately flew the aircraft into the ground are devastating, aircraft-assisted suicide involving commercial flights with passengers on board are rare (Politano and Walton, 2016). It is more common for aircraft-assisted suicide to be carried out by a single occupant pilot in a light aircraft (Lewis et al., 2007). In United States of America, such tragedies account for 3.75% of pilot incapacitations (Lewis et al., 2007), and in Australia, 3.06% annually (Australian Transport Safety Bureau [ATSB], 2007). In their 2015 paper, the Aerospace Medical Association; Ad Hoc Woking Group on Pilot Mental Health. (2012) advocated for assessment of “less severe” and “more common” mental health issues, such as stress and anxiety.

Accurately assessing pilot mental health is challenging. Pilots are renown for underreporting physical and mental health issues so as to not jeopardise their medical, and by extension their licence to fly. Furthermore, pilots’ passion for flying and investment in their career has been suggested to result in a coveting of their licence, (i.e. strong desire to possess and maintain). This in turn results in threats to their licence (i.e. medical issues) becoming a mental health risk (Lewis et al., 2007; Aerospace Medical Association; Ad Hoc Woking Group on Pilot Mental Health, 2015). In a research context, higher prevalence for general anxiety and depression symptoms, along with suicidal thoughts, has been associated with anonymous and questionnaire method assessment (Pasha and Stokes, 2018), whereas database search methodology has tended to yield low prevalence rates for psychological disorders (Ackland et al., 2022). Databases further identify prevalence, albeit low, for other psychological disorders such as obsessive-compulsive disorder (OCD), attention deficit hyperactivity disorder (ADHD), and stress-related disorders to name a few (Ackland et al., 2022). The limitations of the methodology and narrow focus of research into pilot mental health, coupled with a tendency for pilots to be reluctant to report mental health issues complicates the current understanding of pilot mental health.

Importantly, significant stressors increase the risk of mental health issues, as opposed to being solely responsible (Lazarus and Folkman, 1984). This is highlighted in the diathesis-stress model, which illustrates that mental health issues arise due to the interaction of a stressor and an underlying vulnerability, which often occur in the absent of protective factors, rather than as a reaction to a stressor alone (Brannon and Feist, 2007). Hence, and while occupational stressors and life event stressors such as relationship breakdown, age, retirement or other disruptions from flying are often associated with mental health issues (Castelo-Branco et al., 1985; Lewis et al., 2007; Feijó et al., 2012; Politano and Walton, 2016; Cahill et al., 2019), their effect is not universal. Further, and in line with the diathesis-stress model, the stress-buffering hypothesis states that the effect of any vulnerability-stress interaction can be buffered by protective factors, such as coping strategies. In this regard, emotional intelligence and exercise have been associated with better mental health outcomes for pilots (Feijó et al., 2012; Guo et al., 2017). It is, therefore, important to understand that mental health issues do not exist in a vacuum and when investigating pilot mental health, consideration for associated factors such as stressors, vulnerability factors (e.g., personality), and protective factors (e.g., coping strategies) are important.

The occurrence of the COVID-19 pandemic, which developed in Australia in March of 2020, had a particularly devastating effect on the aviation industry globally. In Australia, travel both internationally and interstate was restricted well into 2022 during which time the states also experienced several lockdowns which included closure of occupational and educational institutions, social and exercise clubs, and restriction of movement (i.e., 5 km radius from homes).

Early studies into the impact of the pandemic in the general population found increased depression and anxiety symptoms reported during this time (Sameer et al., 2020). In particular, strict lockdown and quarantine procedures were considered risks to mental health, and were associated with increased depression and anxiety symptoms more so than simply social distancing (Meyer et al., 2020). Additionally, it was identified that the most utilised coping strategies, such as watching television, chores, social networking, eating well, music, sleeping, were unsurprisingly home-based coping strategies, likely due to the restrictions imposed during this time reducing access to outdoor activities and in-person socialising (Sameer et al., 2020). The reduction in physical activity due to COVID-19-related restrictions in particular was associated with poorer mental health for adults in the general population, especially those who were previously physically active rather than sedentary adults (Meyer et al., 2020).

At the outset of the pandemic, Vuorio and Bor (2020) predicted that pilot mental illness and suicides would potentially see an increase as a direct result of COVID-19 pandemic, specifically the economic effects. They suggested that given life-event stress is a particular risk to pilot mental health and pilot suicidality, that the experience of severe life-events during this time should be taken as an indicator of increased risk, along with distress, depression, and reports of hopelessness. They cautioned that the work-related stress is not just owing to the temporary stand-downs and furloughing, but once reinstated will likely include an increase in workload and demands.

Görlich and Stadelmann (2020) studied the depression, anxiety, and stress of cabin crews before and during the pandemic in Germany and found that while depression scores were higher among crews who were stood down/not flying, anxiety scores were higher among crews who were still flying which they attributed to fear of becoming infected with COVID-19, but that could also be associated with feeling job insecurity. Alaminos-Torres et al. (2021) examined the incidence of Spanish pilots’ psychological distress during the pandemic and found a difference between those who continued to fly and those who had been furloughed or unemployed. Almost half of the sample had high (above cut-off) scores indicating psychological distress. Pilots who were remained working, had scores below the cut-off score for psychological distress.

Taken together, the early research out of the pandemic suggested that mental illness increased and that protective factors, most notably lifestyle factors such as exercise decreased. In specific reference to pilots, it was predicted that the pandemic may impact pilot mental health in similar ways to the general population and additionally due to the significant life stress posed by industry instability, job insecurity and uncertainty of rostering, and increased workload.

The aim of the present study is to assess pilot mental health during the COVID-19 pandemic, while addressing the limitations found in previous research on pilots’ mental health by administering a diagnostic interview covering a range of psychological disorders and placing these findings in context through an examination of risk and protective factors. Specifically, the current study aims to assess the prevalence of mental health diagnoses according to self-report and interview-based measures, use validated measures to identify the primary stressors reported by pilots during the pandemic, and assess vulnerability and protective factors associated with mental health outcomes.

## Materials and methods

### Participants

A total of 77 pilots participated in the study after reviewing the participant information and consent forms, outlining confidentiality and voluntary participation. Four participants completed the interview though failed to complete the questionnaire component, and therefore were not included in the dataset. Table 1 outlines the participants’ demographic information, including whether pilots thought they were impacted by COVID-19 restrictions at time of participation.

**TABLE 1:** Demographic information of participants.

Item	Response type	Pilot (*n* = 73)
Gender	Male	58
	Female	14 (19.18%)
	Unanswered	1
Age	Mean	39.70
	SD	10.70
Time in industry (years)	Mean	16.53
	SD	10.93
Total flight hours	Mean	7,702.79
	SD	5,699.99
Recent flight hours (90 days)	Mean	71.50
	SD	55.47
	N/A (zero hours)	27
COVID-19 impact	Yes	64
	Partly	7
	No	2

### Materials

The materials comprised a: demographics questionnaire, the Diagnostic Interview for Anxiety, Mood, and Obsessive-Compulsive and Related Neuropsychiatric Disorders (DIAMOND) semi-structured interview, and seven questionnaires pertaining to mental health or associated factors including passion for flying, personality, stress, coping, and lifestyle. These questionnaires are described in detail below.

### Demographic information

The demographic questionnaire included questions about: age, sex, time in industry, rank, aircraft type, flight hours, as well as mental health history, impact, and reporting. In addition, participants were asked about the extent that the COVID-19 pandemic impacted their employment.

### DIAMOND semi-structured interview

The DIAMOND (Tolin et al., 2013) is a semi-structured diagnostic interview to be administered by a mental health professional. It assesses for 33 DSM-5 disorders and suicidality.

### Health-Promoting Lifestyle Profile II

The Health-Promoting Lifestyle Profile II (HPLII; Walker et al., 1987) was employed to examine lifestyle factors which may be protective factors in the management of mental health. It comprises a 52-item questionnaire which assesses the extent of behaviours or attitudes which may serve to maintain physical and/or mental wellbeing are adopted. Sub-scales include: Health responsibilities, Physical Activity, Nutrition, Spiritual Growth, Interpersonal Responsibilities, and Stress Management. The internal consistency of the full profile has been reported at 0.94, with the subscales ranging from 0.79 to 0.87 (Walker and Hill-Polerecky, 1996).

### Holmes-Rahe Life Social Readjustment Stress Scale

The Holmes-Rahe Life Social Readjustment Stress Scale (SRSS; Holmes and Rahe, 1967) is an instrument designed to identify the incidence of life events which may increase an individual’s risk of a stress-related issue. Total scores of less than 150 points relate to a relatively low amount of life-change and thus a low susceptibility to stress-related issues. A score between 150 and 300 points relates to a moderate amount of life-change and a 50% chance of a stress-related issue. A score above 300 suggests a high amount of life-change and an 80% chance of stress-related issue, according to statistical prediction modelling. Internal consistency of the measure is acceptable (0.72, Lei and Skinner, 1980).

### Work Stress Questionnaire

The Work Stress Questionnaire (WSQ; Holmgren et al., 2009) is a 21-item questionnaire which assesses four categories of work stress, “Indistinct organisation and conflicts,” “individual demands and commitment,” “influence at work,” and “work interference with leisure times,” with the intention to identify people at risk of sick-leave for work-related stress. The WSQ was originally developed on an exclusively female sample but has since been validated on a male sample (Frantz and Holmgren, 2019). In both samples, retest reliability has shown to be acceptable (Holmgren et al., 2009; Frantz and Holmgren, 2019).

### Coping Inventory for Stressful Situations

The Coping Inventory for Stressful Situations (CISS; Endler and Parker, 1999) was employed to examine pilots’ coping strategies as possible protective factors associated with mental health. It is a 48-item questionnaire, comprised of three, 16-item dimensions of coping styles: Task-oriented, Emotion-oriented, and Avoidance-oriented coping. Alpha coefficients have been reported as 0.92, 0.87, and 0.84 for the respective factors (Cook and Heppner, 1997). Scores are summed for each factor to form scale scores with higher scores indicative of a higher use of the respective coping strategy.

### Patient Health Questionnaire 9

The Patient Health Questionnaire-9 (PHQ-9; Kroenke et al., 2001) is a nine-item questionnaire comprised of the nine diagnostic criteria for a depressive episode in the Diagnostic and Statistical Manual of Mental Disorders (DSM-IV-TR). As a commonly administered questionnaire in the literature for pilot mental health, this measure was included as a comparative assessment of psychological symptoms. Internal consistency for the PHQ-9 is reported to be 0.89 (Cronbach’s alpha; Kroenke et al., 2001).

### The Passion Scale

The Passion Scale (Vallerand et al., 2003) was employed to examine pilots’ passion for flying as a potential risk or protective factor associated with mental health. It is a 14-item questionnaire measuring two types of passion: harmonious and obsessive. The internal consistency of the scale is acceptable with Cronbach’s alpha coefficients for harmonious and obsessive passion scales 0.83 and 0.86, respectively (Marsh et al., 2012).

### NEO-Personality Inventory

The NEO-Personality Inventory (NEO-PI-R; Costa and McCrae, 1992) was employed to examine pilot personality as potential risk or protective factors associated with mental health. It is a 240-item measure of the major five domains of personality: Neuroticism, Extraversion, Openness, Agreeableness, and Conscientiousness. Alpha coefficients for the factors range between 0.86 and 0.92 (Costa and McCrae, 1992).

### Procedure

Pilots were recruited through a series of emails and online advertisements by the Australian Federation of Air Pilots (AFAP). Interested pilots contacted the researcher directly and were forwarded further information, including a consent form. Upon providing consent, two links were provided, one to the interview and another for the online questionnaire battery, with instructions as to order of completion. The participant was provided a unique number to connect their responses to allow for deidentifying the participant throughout the study. Questionnaires were hosted on Qualtrics. The DIAMOND interview was conducted using the videoconferencing platform Zoom for all but three participants, who opted for the videoconferencing platform Skype.

The order in which the interview and questionnaires were conducted were counterbalanced to control for order effects. The DIAMOND interview was delivered in its entirety in this study, which took between 45 min and 2 h(average 60 min). The main author (clinical Psychologist) administered all interviews for consistency. However, to ensure accuracy of diagnosis, 10% of interviews were assessed independently by a supervising clinical Psychologist. These interviews were recorded with the explicit consent of the participant. Diagnoses were matched on all cases (i.e., 100% interrater agreement), with one exception where a “not-otherwise specified” diagnosis was coded by main assessor and not coded by the secondary assessor.

### Data analysis

The results of the measures were collected and stored within Qualtrics for the duration of the data collection period and then transferred into Excel for ease of scoring, as per the respective instructions. Total scores for each measure were calculated for each participant number and these results were then transferred into SPSS. The outcome of the DIAMOND interviews (diagnoses, no diagnoses, sub-clinical, or borderline diagnoses) was also entered into SPSS. Descriptive data were inspected and compared for these measures.

The primary analysis was based on the diathesis-stress model relationship between vulnerability factors, stressors, and protective factors, and the psychological symptoms meeting diagnostic threshold on the DIAMOND interview. A binomial logistic regression analysis was conducted with DIAMOND diagnoses/no diagnoses as the dichotomised outcome variable. To best align with a diathesis-stress model, the variables were entered in blocks, as depicted below. NEO factor *t*-scores were entered together as the first block, followed by harmonious and obsessive passion scores, accounting for the vulnerability factors. Stress scores were entered together in the next block, including the Holmes Rahe Life Stress total scores and the scale scores for the work stress questionnaire. Lifestyle questionnaire (HPII) subscale scores and the subscales for the CISS were entered together in the final block to account for coping and protective factors. Finally, a sensitivity analysis was run to control for entering effects where all variables were entered together. This analysis yielded the same result as the block enter method.

## Results

Table 2 displays the means and standard deviations of all measures used in the study for pilots.

**TABLE 2:** Means and standard deviations of measures for pilots.

Measures	Pilots	
	**Mean**	**SD**
PHQ-9	4.27	4.64
Holmes-Rahe social readjusted stress scale	188.56	129.93
HPLII	134.34	19.02
**Subscales**		
Health responsibility	19.45	4.34
Physical activity	21.78	5.31
Nutrition	24.1	4.21
Spiritual growth	22.79	4.59
Interpersonal relations	25.93	4.63
Stress management	20.29	3.85
**CISS**		
**Subscales**		
Task-oriented	59.59	7.95
Emotion-oriented	36.56	9.75
Avoidance-oriented	45.73	10.57
Distraction	20.64	6.15
Social diversion	16.56	4.72
**Passion scale**		
Harmonious passion	5.03	1.27
Obsessive passion	2.26	1.16
**NEO-PI-R**		
Factor scales *t*-scores		
Neuroticism	46.88	11.91
Extraversion	51.41	10.06
Openness	52.87	8.95
Agreeableness	46.69	9.47
Conscientiousness	52.73	11.83
WSQ-scoring is not conducive to means	%	*N*
**Subscales**		
Low influence at work	21.92	16
High indistinct organisation and conflict	57.53	42
High individual demands and commitments	32.88	24
High work interference with leisure	50.68	37

### DIAMOND interview

The prevalence of diagnoseable mental health conditions in the pilot sample was assessed through the DIAMOND interview. Table 3 displays the diagnosable conditions identified along with the number of individuals who met the criteria. As can be seen in this table, 12 pilots had a single diagnosis, and 15 pilots had multiple diagnoses (between 2 and 4). From the 47 diagnoses in the 27 cases, anxiety disorders were most common (14), followed by Attention Deficit Hyperactive Disorders (10), Adjustment Disorders (8), depressive disorders (MDD = 5, Dysthymia = 1), Substance Use Disorders (2), Obsessive Compulsive Disorder (2), Tic Disorder (2), Eating Disorder Not Otherwise Specified (EDNOS) (2), and Trichotillomania Disorder (1). There were also 16 borderline diagnoses, meaning that the reported symptoms did not fully meet the severity level for diagnosis. Similarly, there were 27 subclinical diagnoses meaning that the reported symptoms did not fully meet the criteria for diagnosis. Additionally, there were 26 historical diagnoses which included depressive disorders (23), PTSD (1), and Substance Use Disorders (2).^1^

**TABLE 3:** Number of diagnoses distributed across diagnostic measure.

DIAMOND interview modules	Disorders	Number of cases			
		**Diagnostic**	**Borderline**	**Sub-clinical**	**Historical**
Obsessive-compulsive related disorders	Obsessive-compulsive disorder	2	0	1	
	Body dysmorphic disorder	0	0		
	Hoarding disorder	0	1		
	Trichotillomania and excoriation disorder	1	0		
Anxiety disorders	Social anxiety disorder	2	1		
	Panic disorder	1	0		
	Agoraphobia	1	0		
	Generalised anxiety disorder	9	2	2	
	Specific phobia	1	1		
	Separation anxiety disorder	0	0		
Mood disorders	Persistent depressive disorder (dysthymia)	1	0		1
	Bipolar I disorder	0	0		
	Bipolar II disorder	0	0		1
	Major depressive disorder	5	0	MDE 2	21
	Cyclothymic disorder	0	0		
	Premenstrual dysphoric disorder	Not administered			
Trauma- and stressor-related disorders	Acute stress disorder	0	0		
	Posttraumatic stress disorder	0	0		1
	Adjustment disorder	8	5	3	
Schizophrenia spectrum and other psychotic disorders	Schizophrenia and schizophreniform disorder	0	0		
	Schizoaffective disorder	0	0		
	Delusional disorder	0	0		
Feeding and eating disorders	Anorexia nervosa	0	0		
	Bulimia nervosa	0	0		
	Binge-eating disorder	0	0		
	Avoidant/restrictive food intake disorder	0	0		
	Eating disorder not otherwise specified	2	0		
Somatic symptom and related disorders	Somatic symptom disorder	0	2		
	Illness anxiety disorder	0	0		
Substance-related and addictive disorders	Substance use disorder	2	0		2
Neurodevelopmental disorders	Attention-deficit/hyperactivity disorder	10	4		
	Tic disorder	2	0		
Total Suicide screen		47	16	8	26
		3			5

MDE, major depressive episode.

As can be seen in Table 3, less than ten per cent (*n* = 6, 8.22%) of the total sample reported symptoms which met the criteria for a current depressive disorder. Additionally, approximately one third (*n* = 22 or 30.14%) of the sample reported a history of symptoms which would meet the criteria for a depressive disorder.

### Patient Health Questionnaire 9

Alignment of the PHQ-9 and DIAMOND diagnoses of depression disorders are presented in Table 4. As can be seen in this table, 11 cases were identified as clinical cases by way of scores above 10 on the PHQ-9, and alignment between PHQ-9 and DIAMOND diagnosis of depressive disorders occurred in five of these cases. The 11 PHQ-9 identified cases included four depressive disorder cases identified on the DIAMOND and one case of severe Adjustment Disorder marked by depressive mood. Additionally, there were two cases who only met criteria for anxiety disorders on the DIAMOND, one case who only met criteria for ADHD on the DIAMOND, and two cases who did not have any symptoms meeting diagnoseable criteria on the DIAMOND. Two cases who reported symptoms which met criteria for a depressive disorder on the DIAMOND, did not score above the threshold to be considered a clinical case on the PHQ-9.

**TABLE 4:** Alignment of PHQ-9 (clinical cut-off of 10) and DIAMOND diagnoses of depressive disorders.

ID #	PHQ-9 score	DIAMOND diagnoses	
		**Mood-related**	**Other**
28	14	MDD	Adjustment disorder
40	16	MDD	GAD
54	18	Dysthymia	Social anxiety disorder Substance use disorder
55	10	N/A	N/A
65	10	MDD	GAD
60	13	Invalid	Invalid
84	10	N/A	GAD Phobia
94	21	Severe adjustment disorder with depressed mood	ADHD
96	15	Historical MDD	GAD OCD
111	10	Sub clinical MDD	GAD Trichotillomania ADHD
100	10	N/A	ADHD
49	5	MDD	
113	7	MDD	

### Suicide

Pilots’ responses to PHQ-9 and DIAMOND items related to suicidality were compared.

As can be seen in Table 5, the DIAMOND interview identified three participants with suicidal ideation, however, further assessment through the DIAMOND procedure determined that these thoughts were not indicative of suicide risk as assessment did not find this ideation was accompanied by plans and/or intent to pursue suicide. In contrast, six respondents endorsed current/recent suicidal thoughts on the PHQ-9. As also can be seen in this table, two cases were captured by both measures. One case was identified with the DIAMOND but not the PHQ-9. Two cases identified by the PHQ-9 were not reported during the DIAMOND, and two cases identified by the PHQ-9 reported a history of suicidal thoughts, not current, during the DIAMOND.

**TABLE 5:** Participants who endorsed suicide focused questions on the PHQ-9 and the DIAMOND.

ID #	PHQ-9 thoughts that you would be better off dead, or of hurting yourself	DIAMOND have you ever thought about hurting or killing yourself?
40	More than half the days	Endorsed
47	Several days	Endorsed
54	More than half the days	Endorsed historically
60	Several days	Not endorsed
90	Several days	Not endorsed
96	Several days	Endorsed historically
113	Not at all	Endorsed

### Psychological impairment

Having established the type and prevalence of mental health issues, it was important to determine their perceived impact on performance. Pilots were specifically asked “has your mental health ever impacted your performance.” Twenty-six pilots described how their mental health had impacted their work performance. These explanations were coded and are outlined in Table 6. The most commonly reported impact was on pilots’ cognitive ability such as concentration, feeling less sharp, and distractibility.

**TABLE 6:** Type of impact or impairment caused by psychological symptoms.

Type of impact/impairment	Example	*N*
Cognitive	Distracted, poor concentration, forgetful, less sharp	12
Behavioural	Interpersonal difficulties, out of character responding	3
Emotional/physiological	Low enthusiasm, agitation, low confidence/doubt, sleep issues	5
Performance	Mistakes	2
Absenteeism	Having to take additional time off	4

The PHQ-9 asks the extent to which any of the mood-related symptoms endorsed on the questionnaire created impairment in life domains, in accordance with DSM-5 diagnostic criteria. Of the 58 participants who endorsed any symptoms on the PHQ-9, 38% (*n* = 22) stated they were impaired, finding it *very difficult*, or *somewhat difficult* to function. Notably, 14 of the respondents reporting a level of difficulty, had PHQ-9 scores under the clinical cut-off of 10. Additionally, three of the respondents with scores above 10 denied any impairment.

### Stressors-life events, work place stress, and COVID-19 impact

In terms of pilot stressors and their impact, the results revealed that 98% of the pilot sample were impacted by COVID-19 at the time of the study, with many reporting being stood down (i.e., furlough), on reduced flying duties, or having to change jobs. To understand the broad effects of COVID-19 on the job, pilots were asked “how was your job impacted by COVID-19?” The responses as reported by pilots were coded and are outlined in Table 7. As can be seen from this table, COVID-19 resulted in the majority of the pilots being furloughed with 30% ultimately losing their job or forced to take early retirement.

**TABLE 7:** Impact of COVID-19 reported by pilots.

Type of impact	*N* *
Reduced hours	32
Reduced pay	21
Furlough	67
Redundancy/unemployment	22
Uncertainty	2
Career progression interruption	6
Relocation/base closure	4
Voluntary leave without pay	4
COVID-19 protocols and procedures/increased workload	10
Change of work type/job	6

*More than one response may have been coded from a single participant.

**FIGURE 1:**
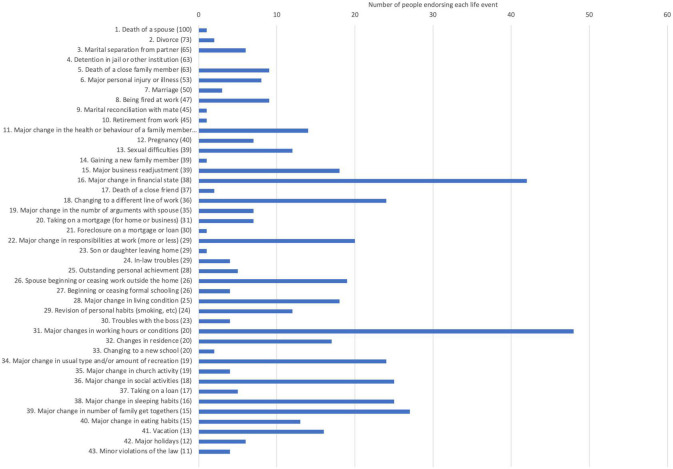
Life events endorsed on the Holmes Rahe Life Stress Inventory.

Pilots also completed The Holmes-Rahe Life Stress Inventory, which examined the accumulative load of stressors that pilots faced in addition to, and including, work-related stressors. The average score on this measure was 189 (SD = 130), which according to the instrument, is aligned to a 50% increased risk of developing a stress-related disorder in the next 2 years. Nine pilots scored above 300 on this instrument, which predicts an 80% likelihood of developing a stress related condition in the next 2 years. Pilots endorsed an average of seven specific life event stressors. As depicted in Figure 1, the most commonly endorsed life events were: “Major change in working hours or conditions,” “Major change in financial state,” “Major change in the number of family get togethers,” “Major change in sleeping habits,” and “Major change in social activities.”

The results emphasise that during the time of the study in the context of a global pandemic, pilots not only experienced increased stressors, but also a change in the extent to which they could engage with valuable support networks, presumably due to COVID-related restrictions and lockdowns. Whether life event stressors (Holmes-Rahe Life Event Inventory) or work-related stressors (Work Stress Questionnaire) differed between groups (diagnoses compared to no diagnoses) was examined by a series of *t* tests. No differences were found between the two groups on these scales [largest *t*, *t*(70) = 1.10, *p* = 0.314 for Life Stress], suggesting that both groups were equally affected by work and life stress during the study, and stressors alone could not account for the differences in mental health conditions.

### Associated factors: vulnerability and protective factors

Associated factors were investigated to determine to what extent vulnerability and protective factors were associated with pilot mental health. The results are presented below.

#### Passion

The Passion Scale was used to assess pilot passion and revealed that pilots have high levels of harmonious passion, with some also having high levels of obsessive passion. Using diagnoses based on DIAMOND, two *t* tests were conducted to examine differences between the “Diagnoses” and “No Diagnoses” groups, in both harmonious and obsessive passion. The results failed to reveal a statistical difference between the two groups for Harmonious Passion [*t*(70) = 0.57, *p* = 0.568]. However, a statistical difference was revealed for Obsessive Passion [*t*(37.87) = 3.03, *p* = 0.003]. Pilots with a diagnosis scored higher on the Obsessive Passion scale than pilots without a diagnosis; the difference in mean representing a large effect size (Cohen’s *d* = 0.829).

#### Personality

The NEO-PI-R was administered and examined alongside NEO provided means (Costa and McCrae, 1992) to identify any distinct characteristics. NEO factor scores are displayed in Table 8 for both the pilot sample and the comparison sample provided. As can be seen in this table, pilots’ scores were different from population norm comparison for Neuroticism (lower), Openness (higher), and Agreeableness (lower). No differences were observed for extraversion or conscientiousness.

**TABLE 8:** Pilot sample mean and comparison mean for NEO factor scores.

NEO factor	Sample mean (SD)	Comparison mean (SD) (Costa and McCrae)	Difference
Neuroticism	72.4795 (25.26)	79.1 (21.2)	6.62055
Extraversion	111.9863 (18.51)	109.4 (18.4)	2.58630
Openness	115.5616 (15.48)	110.6 (17.3)	4.96164
Agreeableness	119.2740 (15.57)	124.3 (15.8)	5.02603
Conscientiousness	127.9041 (20.82)	123.1 (17.6)	4.80411

The extent to which any personality features were associated with psychological diagnoses was subsequently examined to understand what vulnerability factors attributed to personality may be related to psychological symptoms. Independent samples *t* tests were performed using the NEO-PI-R scores, compared between the Diagnoses and No Diagnoses groups. These group differences are displayed in Table 9, with significant differences observed between the “Diagnoses” and “No Diagnoses” groups (see Table 10). Specifically, the “Diagnoses” group had higher Neuroticism, lower Agreeableness, and lower Conscientiousness scores than the “No Diagnoses” group.

**TABLE 9:** Comparison NEO factor scores (mean) between sample and comparison groups.

NEO factor	Sample	Diagnoses	No diagnoses	General population
Neuroticism	72.48 (25.26)	83.48 (25.75)	65.51 (22.84)	79.1 (21.2)
Extraversion	111.99 (18.51)	110.78 (22.54)	113.20 (15.71)	109.4 (18.4)
Openness	115.56 (15.48)	119.04 (15.33)	113.84 (15.33)	110.6 (17.3)
Agreeableness	119.27 (15.57)	112.07 (15.41)	124.07 (13.81)	124.3 (15.8)
Conscientiousness	127.90 (20.82)	120.44 (24.64)	132. 38 (17.20)	123.1 (17.6)

**TABLE 10:** Results of *t* tests on pilot personality factors for diagnoses and no diagnoses groups.

Neo factor	*t*	*df*	Confidence interval		*p*	Cohen’s *d*
			**Lower**	**Upper**		
Neuroticism	3.081	70	6.33577	29.60497	0.003	0.750
Extraversion	0.491	41.283	−12.37628	7.53183	0.626	0.131
Openness	1.388	70	−2.26796	12.65315	0.169	0.338
Agreeableness	3.415	70	−18.99728	−4.98790	0.001	0.831
Conscientiousness	2.417	70	−21.78183	−2.08483	0.018	0.588

To understand whether the differences found between groups were also different compared to the population comparison means, further *t* test were run with an adjusted alpha of 0.025 to control for familywise error. The results are displayed in Table 11. As can be seen in this table, the resulting personality profiles of the groups were very different; where the “Diagnoses” group show significantly higher Openness and lower Agreeableness compared to the General Population. The “No Diagnoses” group differed from the General Population with significantly lower Neuroticism and higher Conscientiousness. Moderate effects sizes (Cohen’s *d* = 0.539–0.595) were found for these differences, with a large effect size found for the “Diagnoses” group Agreeableness (Cohen’s *d* = 0.793).

**TABLE 11:** Results of *t* tests between study groups and general population.

NEO factor	*t*	*df*	Confidence interval		*p*	Cohen’s *d*
			**Lower**	**Upper**		
**Neuroticism**						
Diagnoses	0.884	26	-5.8046	14.5676	0.385	0.170
No diagnoses	3.991	44	-20.4517	-6.7261	0.000	0.595
**Extraversion**						
Diagnoses	0.318	26	-7.5399	10.2954	0.753	0.061
No diagnoses	1.623	44	-0.9189	8.5189	0.112	0.242
**Openness**						
Diagnoses	2.842	26	2.3347	14.5393	0.009	0.547
No diagnoses	1.420	44	-1.3615	7.8504	0.163	0.212
**Agreeableness**						
Diagnoses	4.123	26	-18.3218	-6.1301	0.000	0.793
No diagnoses	0.113	44	-4.3836	3.9170	0.910	0.017
**Conscientiousness**						
Diagnoses	0.560	26	-12.4026	7.0915	0.580	0.108
No diagnoses	3.618	44	4.1100	14.4456	0.001	0.539

In summary, whereas pilots as a whole had lower neuroticism than population norms, unsurprisingly, pilots with diagnoses had higher neuroticism than pilots with no diagnoses. Neuroticism scores for the “Diagnoses” group was considered in line with the general population, however, and it was the lower scores for the “No Diagnoses” group which were significantly different with a moderate effect size. Similarly, pilots as a whole were lower in agreeableness, however further examination revealed that this was mainly the “Diagnoses” group which significantly differed from the general population mean with a large effect size, whereas the “No Diagnoses” group means for agreeableness were in line with population comparison.

While overall, pilot conscientiousness was not significantly different to population norms, the “Diagnoses” and “No Diagnoses” groups differed on conscientiousness, with the respective scores also notably different (though in different directions) from population norms for conscientiousness. Conscientiousness scores for pilots in the “No Diagnoses” group were significantly higher than general population comparison, with a moderate effect size, while the “Diagnoses” group conscientiousness scores were considered in line with population norms. High openness was found for pilots as a whole, however when the two groups were compared to the comparison group, significant differences were found between the “No Diagnoses” and comparison group, but not between the “Diagnoses” and comparison group.

### Protective factor: coping styles and lifestyle

**FIGURE 2:**
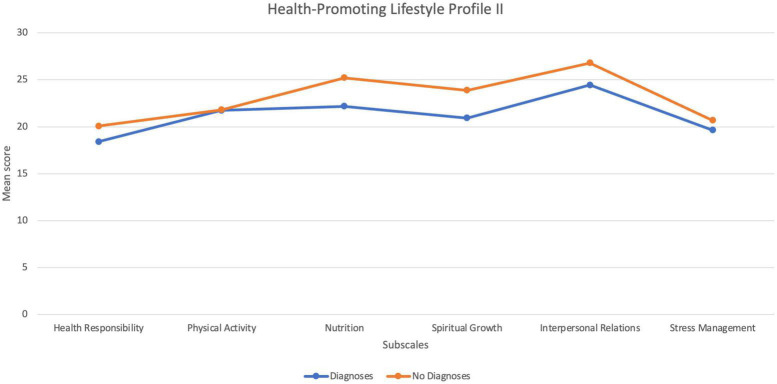
Scores between groups on HPLII.

Scores on the CISS reflected a tendency for pilots to take a primarily Task-Oriented coping style, followed by Avoidant Oriented Coping, and Emotion Oriented Coping. In addition to intentionally used coping strategies, the Healthy Lifestyle Profile II was administered to understand whether any lifestyle attributes may be protective for pilots’ psychological health. The scores on the six subscales for this measure, for the pilots with diagnoses and the pilots without diagnoses are displayed in Figure 2. As can be seen in this figure, these groups mainly differ on subscales pertaining to nutrition, spiritual growth (actualisation), and interpersonal relations. The results of a series of *t* tests illustrated statistical differences for these three subscales, as outlined in Table 12, with moderate effect sizes.

**TABLE 12:** Results of *t* tests for HPLII between groups.

Subscales	*t*	*df*	Confidence interval		*p*	Cohen’s *d*
			**Upper**	**Lower**		
Health responsibility	1.519	70	−0.17695	0.11650	0.133	0.370
Physical activity	0.011	70	−0.00185	0.16512	0.991	0.003
Nutrition	3.169	70	−0.34239	0.10804	0.002	0.771
Spiritual growth	3.027	70	−0.39537	0.13060	0.003	0.737
Interpersonal relations	2.194	70	−0.26914	0.12267	0.032	0.534
Stress management	1.231	70	−0.14352	0.11390	0.213	0.300

### Diathesis-stress model

With the vulnerability and protective factors in mind, the study sought to determine which of the factors included in the study predicted association with a psychological diagnosis when analysed together, in line with a diathesis-stress model. Hence, a binomial logistic regression was conducted. DIAMOND “Diagnoses” or “No Diagnoses” was the dichotomised outcome variable. Independent variables were entered in blocks with NEO factor scores entered as Block 1, followed by Passion scores as Block 2, Stress scores (Holmes Rahe Life Stress Inventory, Work Stress Questionnaire) as Block 3, and protective factors (HPLII, CISS) as Block 4.

The results of the binomial logistic regression analysis revealed that the independent variables of NEO Agreeableness, Obsessive Passion, and Nutrition were significant predictors of group membership. At the first step, Agreeableness correctly predicted 65.3% overall (*p* = 0.003). This improved to 75% with the addition of Obsessive Passion at the second step (*p* = 0.020), and then finally to 79.2% with the addition of nutrition (*p* = 0.049). Stressors added in the third block did not meaningfully contribute to the model and so were not included in the equation. The final model is shown in Table 13.

**TABLE 13:** Results of logistic regression analysis showing factors associated with pilot group membership.

Predictors	*B*	SE	Wald test	No diagnoses				Diagnoses		
				***p***	**OR**	**95% CI OR**		**OR**	**95% CI OR**	
						**Lower**	**Upper**		**Lower**	**Upper**
NEO agreeableness	0.058	0.036	2.663	0.103	1.060	0.988	1.137	0.943	0.880	1.012
Obsessive passion	-0.100	0.042	5.643	0.018*	0.905	0.833	0.983	1.105	1.017	1.200
Nutrition	1.443	0.732	3.890	0.049*	4.234	1.009	17.770	0.236	0.056	0.991

OR, odds ratio; CI OR, confidence interval for odds ratio.

*p < 0.05.

The overall model accuracy was greater for the “No Diagnoses” group than for the “Diagnoses” group, possibly due to the lower number of participants in the “Diagnosis” group. This model correctly predicted 66.7% of those with diagnoses and 86.7% without diagnoses, with an overall success rate of 79.2%. The success of the model remained with a sensitivity analysis (i.e., variables entered together in one block rather than four) revealing no differences with the first analysis. These results indicate that membership in the “Diagnoses” group is associated with lower agreeableness, higher obsessive passion, and lower nutrition.

Specifically, a one unit change in agreeableness will increase the odds of “No Diagnoses” by 6% relative to the odds of diagnoses. Similarly, a one unit change in nutrition will increase the odds of “No Diagnoses” by 423% relative to the odds of a diagnosis. In contrast, a one unit change in “obsessive passion” will increase the odds of diagnoses by 10% relative to the odds of no diagnoses.

## Discussion

This study aimed to comprehensively assess the prevalence and nature of pilot mental health, as well as associated vulnerability and protective factors during the COVID-19 pandemic. This research addresses limitations of the previous literature on pilot mental health and is of importance due to the unique roles pilots have and stressors they face, as well as the pursuit of safety within the aviation industry. The results revealed that pilots can, and do develop psychological disorders, with over one third of the sample meeting criteria for at least one DIAMOND diagnoseable disorder. The majority of these cases met criteria for a single diagnosis, which has a better prognostic value than multiple disorders (Britt et al., 2018).

The identified disorders varied in both nature and severity, with the most commonly identified diagnosable disorders being anxiety disorders (19.18%). It is important to discuss that there is notable variability between anxiety disorders and there are other disorders which are characterized by anxiety, but that are diagnostically separated from the anxiety disorders, such as OCD and PTSD. Each of these disorders differ in symptomatology, the pervasiveness of their symptoms and the extent that an individual may be impaired by these symptoms (American Psychiatric Association [APA], 2013). Often, the distinction between these disorders is not reflected in pilot studies that have assessed *anxiety* (Ackland et al., 2022). In this study, four different anxiety disorders were identified (generalised anxiety disorder, social anxiety disorder, specific phobia, and panic disorder/agoraphobia), in addition to OCD. Anxiety disorders can be relatively straight forward to treat and the associated difficulties can be contained to acute “triggers” rather than having a pervasive effect (Andrews et al., 2002). In this way, it is entirely possible for an anxiety disorder to be separate from, and cause no safety concern for flying. However, this is not necessarily the case for all anxiety disorders (i.e., Panic Disorder; Marsh et al., 2010), emphasising the importance of a proper diagnostic understanding of pilots’ anxiety related concerns.

Attention Deficit Hyperactivity Disorder was the second most identified “disorder” in the current study (13.70%), a result that is not reflected in other studies (see Ross et al., 2007, Laukkala et al., 2017). However, it is believed that many cases of ADHD go undiagnosed, mostly because the “disorder” does not necessarily manifest in problematic or impairing ways to attract diagnosis (Sedgwick et al., 2019). Indeed, ADHD can provide certain strengths, most notably in the areas of hyper-focus, reactivity, and problem solving. For pilots, such strengths could be complimentary with a flying career (Swann, 2021). However, possible difficulties associated with ADHD may be considered especially challenging particularly in an airline role, such as executive dysfunction resulting in poor concentration and organisation (American Psychiatric Association [APA], 2013). Most regulatory agencies worldwide consider ADHD a disqualifying condition (Laukkala et al., 2017). Further, stimulant medication which is frontline treatment for many with ADHD is prohibited (Morse and Bor, 2006; Swann, 2021), despite its efficacy in ameliorating many of the challenging symptoms of ADHD, including those which have been associated with accidents in motor vehicles (Chang et al., 2014). The perceived incompatibility between ADHD and flying as well as the restrictions around effective treatment options likely further disincentivise pilots reporting or seeking assessment of ADHD symptoms, beyond usual reluctance toward psychological reporting.

Depressive disorders were not as commonly found as anxiety disorders and ADHD in the current study. Six cases of depressive disorders were found, accounting for approximately 8% of the sample. This rate is still higher than the incidence rate in the general population (WHO prevalence rates 4.6%, World Health Organization [WHO], 2017), however, not necessarily in the context of the pandemic where higher rates of depression have been identified in the general population (Sameer et al., 2020). It is also important to remember that this study was cross-sectional in nature not longitudinal and, therefore, inadequate to suggest that just because depressive diagnoses were not identified at the time of the study that these symptoms would not emerge at a later date.

There were five cases of reported thoughts of suicide (as well as an additional two reported on the PHQ-9 but not the DIAMOND), however there were no cases of risk, determined by appropriate suicide risk procedure as outlined in the DIAMOND module administered to each participant. This discrepancy between reported suicidal thoughts and suicide risk is not at all uncommon, as discussed by Klonsky and May (2014), and places previous studies’ reports of suicidality based entirely on endorsement of thoughts in question. This is further highlighted by the fact that pilot suicide, and particularly aircraft assisted suicide is still extremely rare especially in commercial aviation, despite studies reporting suicidal ideation in over 12% of their sampled pilots (Wu et al., 2016; Cahill et al., 2019). There are purported key differences which distinguish between suicide ideators and those who go on to attempt suicide. These differences include problems with premeditation (thinking through consequences before action), a history of self-injury, violence, low social support, and alcohol use (Klonsky and May, 2014). While these characteristics are atypical in the commercial pilot population, the potential for them to be both present but also identifiable is another example of the need for a more detailed assessment of pilots’ mental health at the point of licence renewal, and a caution against making presumptions based on limited reported symptoms.

Although the PHQ-9 is a common questionnaire used in pilot mental health studies, in the present study, the PHQ-9 as a screening tool was found to be inadequate for the purpose of gaining an overall picture of pilot psychological health. This was primarily due to its focus on depression and suicide which, as discussed, was only a small proportion of possible difficulties pilots may face. Further, the PHQ-9 was not entirely accurate in identifying all depressive disorder cases identified using the DIAMOND interview. The cases “missed” by the PHQ-9 are a concern, and highlight that the high face validity of questionnaires may facilitate reverse malingering. There were similarly cases identified on the PHQ-9 and not the DIAMOND. This could be due to symptoms identified on the DIAMOND that were ruled out as a clinical case through further clarification; an advantage of clinical assessment via interview. However, as all psychological inquiry can be manipulated and impression managed due to relying on self-report, this could also point to impression management by interview respondents. Nevertheless, the DIAMOND interview was able to extend the assessment of mental health issues and identify 16 pilots’ mental health issues that were not apparent through PHQ-9 administration.

It is often presumed that the presence of a psychological disorder necessarily results in impairment or that certain disorders are more severe, resulting in more impairments than others. In the current study, the inaccuracy of these presumptions is highlighted in the findings of subthreshold PHQ-9 cases which reported symptoms as impairing, and some threshold cases which reported little or no impairment. When psychological symptoms were reported to impact a pilot, the nature of impairments varied and included cognitive (e.g., distracted and forgetful), behavioural (e.g., interpersonal difficulties), emotional (e.g., agitated, low confidence, and low enthusiasm), and performance impairments (e.g., making mistakes), as well as absenteeism; with only performance and cognitive impairments likely to have specific safety implications on flight. Overall, it is clear that impairment, and/or the degree of impairment is as idiosyncratic as most other aspects of mental health. Increasing the understanding and support of pilot mental health particularly with regard to the point that the presence of a symptom, or even a disorder, does not equate to impairment necessarily, and performance impairment specifically, may lower pilots’ own fear and reluctance to report and seek help for mental health issues.

At the time of the study, the global pandemic had wide-reaching impact and caused particular stress within the aviation industry (Görlich and Stadelmann, 2020; Vuorio and Bor, 2020). Unsurprisingly, the most commonly endorsed stressor on the SRSS in the current study was work-related changes (hours or conditions), followed by financial changes. Pilots’ stress scores on average (189) placed pilots at, at least a 50% increased likelihood of a stress-related health issue in the coming 2 years. After “changes in working hours or conditions” and “financial state,” pandemic pilots reported “major change in family get togethers” and “major change in social activities” as the most common life event changes, highlighting that in addition to the increased work-related stressors, pilots were not able to engage with valuable protective factors to manage their stress.

Previous research has linked life stressors to pilot suicidality (Lewis et al., 2007). This was not substantiated in this study. However, the current study stressor/s are not the types of life stressors identified in the previous research. Life event stressors identified in case studies of aircraft-assisted pilot suicide were often events which would put an end to the pilot’s flying career, such as criminal charges, or physical health deterioration. Even though the pandemic impact is similar in that it disrupts pilots’ ability to fly, there is still every expectation that this would be a temporary disruption. Nevertheless, the findings taken together depict the extent to which a pilot’s inability to fly (i.e., removed from aspects of having employment and financial stability) may be integral to their psychological wellbeing.

Despite pilots’ high stress scores and numbers of stressors reported, stressors on their own did not explain the incidence of psychological difficulties experienced by pilots in the current study. Stress scores were high for the entire sampled group, with the stress scores and number of stressors endorsed the same for both diagnoses and no diagnoses pilot groups. This finding is consistent with the diathesis-stress model which emphasises that it is an interaction between stressors and underlying vulnerability factors, possibly buffered by certain protective factors, which results in psychological issues.

In exploration of associated factors beyond stressors, the current study identified specific vulnerability and protective factors associated with pilot mental health. Specifically, the current study identified that pilots’ personality, obsessive passion, and lifestyle habits were associated with the incidence of psychological disorder in the context of pandemic stressors.

With regard to “passion,” pilots in the Diagnoses group scored higher on obsessive passion compared to pilots in the No Diagnoses group, which is in line with the dualistic model of passion outlining obsessive passion as psychologically problematic (Vallerand et al., 2003). This illustrates how pilots who position flying as a hallmark of their identity can suffer adverse psychological outcomes when their ability to fly is disrupted, such as was the case during the present study due to COVID-19.

The present study identified an average profile of pilots’ personality which differed from population norms (Costa and McCrae, 1992) with comparatively low neuroticism, high openness, high agreeableness, and high conscientiousness. When the pilot sample was divided into groups based on current diagnoses, two distinct personality profiles emerged which differed in reference to comparison group norms. An examination of the personality profiles for these groups revealed that pilots with a diagnosis had higher neuroticism, lower conscientiousness, higher openness, and higher agreeableness than pilots without a diagnosis. In the general population, high neuroticism and low conscientiousness is associated with greater psychological vulnerability (Malouff et al., 2005). As such, it may not seem surprising that those pilots with higher trait neuroticism and lower trait conscientiousness have greater likelihood toward psychological disorder. However, this is not an entirely accurate depiction of how the neuroticism and conscientiousness scores differed between groups with respect to the general population. While pilots with a diagnosis did have higher neuroticism scores compared to pilots without a diagnosis, the scores were in line with the general population, meaning that their neuroticism scores were not higher than “normal.” Rather, the pilots without a diagnosis had significantly *low* neuroticism scores compared to the general population.

Similarly, while pilots with a diagnosis had lower conscientiousness scores compared to pilots without a diagnosis, these scores were also in line with the general population, and this difference was explained by the pilots without a diagnosis who had significantly *high* conscientiousness scores compared to the general population. These are important distinctions as the implication of this is that pilots with high neuroticism and low conscientiousness will not present this way with reference to the general population, they will only appear to differ in these ways with reference to other pilots.

In summary, with respect to the general population, pilots with a diagnosis were more agreeable and more open than pilots than pilots without a diagnosis. Conversely, pilots without a diagnosis had low neuroticism and high conscientiousness when compared to the diagnosis group and with respect to the general population.

On the CISS measure, pilots’ tendency to use task-orientated coping strategies emerged. However, in addition to intentionally applied coping strategies, pilots’ results on the lifestyle inventory, demonstrated high scores across all domains, with interpersonal growth, nutrition, and spiritual growth, the highest. Spiritual growth on the HPLII relates to achievement striving and as such is not a surprising finding in the context of pilots. It is, however, perhaps a commonly overlooked lifestyle domain which can have positive psychological benefits. In this regard, it could be likened to the human need toward self-actualisation (Maslow, 1943). Interpersonal relations followed by nutrition were the most endorsed lifestyle factors on the HPLII for all pilots on average, though these were more endorsed by pilots in the “No Diagnoses” group. This identified that pilots without diagnoses engaged in more interpersonal activities and ate more healthily than pilots in the diagnoses group. This suggests that pilots’ lifestyle habits do potentially protect against, or buffer the experience of, psychological symptoms.

Pilots overall scored lowest on health responsibility, a scale measuring the extent to which pilot seek medical attention for their general health. This could be owing to pilots having generally better health and annual medical check-ups for their licence requirements, and as such less need for interim medical attention. However, this is also in line with literature describing pilots’ tendency to “reverse malinger” to not jeopardise their ability to continue flying, as well as previous research which showed pilots attending their general practitioner far less than the general population (Sykes et al., 2012).

From a theoretical perspective, the results of this study supported a diathesis-stress model for pilot mental health. That is, that specific vulnerability factors were identified to explain the effect of stress on pilots with and without mental health issues. A regression analysis was conducted to explore the total impact of the vulnerability and protective factors in relation to pilot psychological diagnoses. Of all the factors for which differences were found between the “Diagnoses” and “No Diagnoses” groups, three factors emerged as significant in this model: agreeableness, obsessive passion, and nutrition. The results of the regression, therefore, suggest a model whereby trait agreeableness and obsessive passion may create vulnerability in a pilot, who, if then experiences life stressors, will be more likely to develop a psychological disorder. The results of this analysis further suggest that of possible coping strategies and lifestyle factors, nutrition was the most important protective factor, which could possibly buffer the effects of vulnerability-stress interactions for pilots without a diagnosis.

### Limitations and future research

This study, though comprehensive, is not without its limitations. Because the data were collected entirely within the COVID-19 global pandemic, these findings exist within the context of significant industry disruption. Nevertheless, COVID-19 is not the first of such disruptions, nor is it expected to be the last.

In addition, this study only measured above threshold disorders based on the DIAMOND interview, however there is a valid argument that difficulties be identified at the symptom level, especially in aviation. Future research may well be valuable to investigate the prevalence and impact where symptoms are not yet reaching the severity of a diagnoseable disorder or lack some of the symptoms to meet full criteria for a diagnoseable disorder, or the symptom cluster are atypical for any specific disorder.

Additional limitations pertain to the sample, and include the exclusive recruitment of pilots through an Australian pilot association, which likely resulted in an almost entirely Australian pilot population. As the extent to which such a population would be representative of pilots globally is unknown, future research should expand this pilot base in order to determine if the same findings (mental health conditions and associated factors) are present in the wider pilot population. In addition, and with a larger sample, future research could investigate the differences between pilots based on type of operation (regular public transport vs. GA), fixed-wing vs. rotary wing operations, international vs. domestic operations, and rank within organisation.

Finally, this study collected cross-sectional data which may not adequately represent the extent to which pilots may develop issues over time. A longitudinal study in this area would provide important insight about the development of mental health issues, as well as the impact associated risk and protective factors have on the development of these issues.

## Conclusion

In conclusion, this study conducted during the global pandemic highlighted the incidence of pilot health issues. Anxiety Disorders, ADHD, Adjustment Disorders, followed by Depressive Disorders were the most commonly found disorders, based on the DIAMOND interview. Pilots reported high levels of stress during the pandemic, placing them at an increased risk for the development of stress-related health conditions, however stress alone did not explain the difference between those pilots who met criteria for a diagnosis compared to those pilots who did not. Rather, pilot personality, passion, and lifestyle factors emerged to explain this difference. In particular, and supporting the diathesis-stress model for pilot mental health, trait neuroticism, obsessive passion, and nutrition provided the most meaningful contributions to the model of pilot mental health. This research provides a more comprehensive assessment of pilot mental health than previous research which has relied on questionnaire or database search methods and has somewhat neglected investigating broad risk and protective factors. Further, this research demonstrates that pilots will report mental health symptoms, even if limited to the research context under conditions of confidentiality, providing a promising precedent for future research to also explore pilot mental health more thoroughly. Given the relatively high rate of diagnoseable mental health conditions identified in this study, it may be pertinent to revisit the licencing requirements for pilots with regard to mental health conditions, with more of a focus on impairment and less on diagnosis. The discrepancy between reported symptoms and related experience of impairment emphasises that there should not be a presumption that pilots’ mental health issues are incompatible with flight safety. In fact, while pilots reporting mental health issues is in the best interest of safety, especially if the alternative is pilots suffering in silence, a mental health diagnosis should not be considered limiting for commercial operations. Rather, the focus should be on assessing the extent to which a pilot is impaired in their duty and supporting to ameliorate this impairment. Further, given that symptoms and associated impairments fluctuate, the industry would be advised to take a more adaptive and flexible position with regard to mental health, in contrast to the present dichotomous approach.

## Data availability statement

The datasets presented in this article are not readily available because requirements set by the Ethics Committee. Requests to access the datasets should be directed to BM, b.molesworth@unsw.edu.au.

## Ethics statement

The studies involving human participants were reviewed and approved by the UNSW Ethics Panel. The patients/participants provided their written informed consent to participate in this study.

## Author contributions

BM created the research presented in the manuscript. BM designed the study in collaboration with CA and JG. CA recruited the pilot and conducted the clinical interview under the supervision of BM and JG. BM and CA analysed the data. CA produced a draft version of the manuscript, and amended and edited by BM and JG. All authors contributed to the article and approved the submitted version.

## Footnotes

1 DIAMOND interview does not have provision to assess historical diagnoses for all modules. Historical diagnoses are only assessed within the depressive disorders, stress-related disorders, substance use disorders and panic disorder.
